# A Review of Spatial Variation of Inorganic Nitrogen (N) Wet Deposition in China

**DOI:** 10.1371/journal.pone.0146051

**Published:** 2016-01-05

**Authors:** Lei Liu, Xiuying Zhang, Shanqian Wang, Xuehe Lu, Xiaoying Ouyang

**Affiliations:** 1 Jiangsu Provincial Key Laboratory of Geographic Information Science and Technology, International Institute for Earth System Science, Nanjing University, Nanjing, 210023, China; 2 Jiangsu Center for Collaborative Innovation in Geographical Information Resource Development and Application, Nanjing, 210023, China; 3 State Key Laboratory of Remote Sensing Science, Institute of Remote Sensing and Digital Earth, Chinese Academy of Sciences, Beijing, 100101, China; CAS, CHINA

## Abstract

Atmospheric nitrogen (N) deposition (N_dep_), an important component of the global N cycle, has increased sharply in recent decades in China. Although there were already some studies on N_dep_ on a national scale, there were some gaps on the magnitude and the spatial patterns of N_dep_. In this study, a national-scale N_dep_ pattern was constructed based on 139 published papers from 2003 to 2014 and the effects of precipitation (P), energy consumption (E) and N fertilizer use (F_N_) on spatial patterns of N_dep_ were analyzed. The wet deposition flux of NH_4_^+^-N, NO_3_^-^-N and total N_dep_ was 6.83, 5.35 and 12.18 kg ha^-1^ a^-1^, respectively. N_dep_ exhibited a decreasing gradient from southeast to northwest of China. Through accuracy assessment of the spatial N_dep_ distribution and comparisons with other studies, the spatial N_dep_ distribution by Lu and Tian and this study both gained high accuracy. A strong exponential function was found between P and N_dep_, F_N_ and N_dep_ and E and N_dep_, and P and F_N_ had higher contribution than E on the spatial variation of N_dep_. Fossil fuel combustion was the main contributor for NO_3_^-^-N (86.0%) and biomass burning contributed 5.4% on the deposition of NO_3_^-^-N. The ion of NH_4_^+^ was mainly from agricultural activities (85.9%) and fossil fuel combustion (6.0%). Overall, N_dep_ in China might be considerably affected by the high emissions of NO_x_ and NH_3_ from fossil fuel combustion and agricultural activities.

## Introduction

Atmospheric nitrogen (N) deposition (N_dep_) has dramatically increased in the past few decades owing to the rapid increases of industrialization, urbanization and intensified agricultural production in China [1–4]. Currently, the intensity of N_dep_ is equal or even exceeds that in Europe and America [5], causing general concerns of the governments and the public. Increased N_dep_ in terrestrial or aquatic ecosystems or both degrade human health [6], alter chemical components of soil and water [7], influence greenhouse gas balance [8] and reduce biological diversity [9]. Therefore, it is critical to estimate N_dep_ patterns for quantifying the effects of N amendment and establish control measures to improve environmental quality.

Some studies have reported the observed results of N_dep_ at a local scale in China [10–12]. These investigations mainly collected N deposition samples from different sampling sites in some local areas, determined the fluxes of N_dep_, characterized the seasonal or annual variation, assessed the potential ecological risk and analyzed possible sources of N_dep_ [1, 13–19]. They have demonstrated that atmospheric N_dep_ in China increased rapidly over recent decades primarily due to increased energy consumption and N fertilizer use, and this increasing trend will continue in the future with the continuing development of China's economy. However, most of these studies did not give the magnitude and spatial pattern of N_dep_ throughout China due to the difficulty of obtaining the N fluxes on a large area of China [20–24].

There have been several studies on N_dep_ throughout China. For example, Lu and Tian [1] reported N_dep_ peaked over central south of China, with an average value of 12.89 kg ha^-1^ a^-1^ from site-network observations. Moreover, they [14] resulted in the N_dep_ was 14.05 kg ha^-1^ a^-1^ (on the assumption that wet N_dep_ contributes 70% of bulk deposition) in the recent decade, combining site-level monitoring and atmospheric transport model, and they resulted that the most rapid increase centered in southeastern China. Liu et al. [3] believed that N_dep_ increased to 21.1 kg ha^-1^ a^-1^, based on the atmospheric deposition monitoring network and the published papers, and they pointed out that the N_dep_ in the industrialized and agriculturally intensified regions of China as high as the peak levels in northwestern European in 1980s. Jia et al. [25] concluded that N_dep_ was 13.87 kg ha^-1^ a^-1^ in the 2000s, using the N fluxes at 41 stations, with an increasing rate of 25% than that in the 1990s and the highest N_dep_ occurred in southern China. Zhu et al. [4] demonstrated that N_dep_ was 13.18 kg ha^-1^ a^-1^, accounting for 73% of total N_dep_ and peaked in central and southern China.

From the above analysis, the magnitude of N_dep_ and the spatial distribution of N_dep_ were not consistent in the mentioned studies. Liu et al. [26] believed that Zhu et al. [4] might underestimate the dissolved inorganic nitrogen (DIN) due to the uncertainty resulting from the sampling, storage and analysis methods in their study [26]. Pan and Li [27] thought that Lu and Tian [14] underestimated N_dep_ based on a ratio of 0.7 and found the ratio was about 0.4 in Northern China [28]. Therefore, it is still an open question on the spatial pattern and magnitude of N_dep_ in China.

On the national scale of N_dep_, the influencing factors on the spatial variations of N_dep_ were also studied. The spatial variations of N_dep_ had been greatly influenced by factors including N fertilizer use (F_N_), energy consumption (E), and precipitation (P). Zhan et al. [29] hold that F_N_, E, and P jointly explained 84.3% of the spatial pattern of N_dep_, of which F_N_ (27.2%) was the most important, followed by E (24.8%) and P (9.3%). Zhu and He [4] found P and F_N_ can explain 80–91% of the spatial variation of N_dep_, but E had little effect on this variation. Jia et al. [25] reported that F_N_, E and P combined contributed 79% on the spatial variation of N_dep_, while E contributed 80% of decadal variation followed by F_N_, but P had little effect. These results obtained different opinions on the influences of F_N_, E and P on the spatial variations of N_dep_. The interrelationship between N_dep_ and these factors also should be further studied on a national scale.

The present study aims to (1) identify the magnitude and the spatial pattern of N_dep_ throughout China, (2) summarize how precipitation, N fertilizer use and energy consumption influencing spatial pattern of N_dep_, quantify the correlation between factors and N_dep_, and (3) determine the contributions of potential sources to the magnitude of N_dep_ in China.

## Materials and Methods

**Fig 1:**
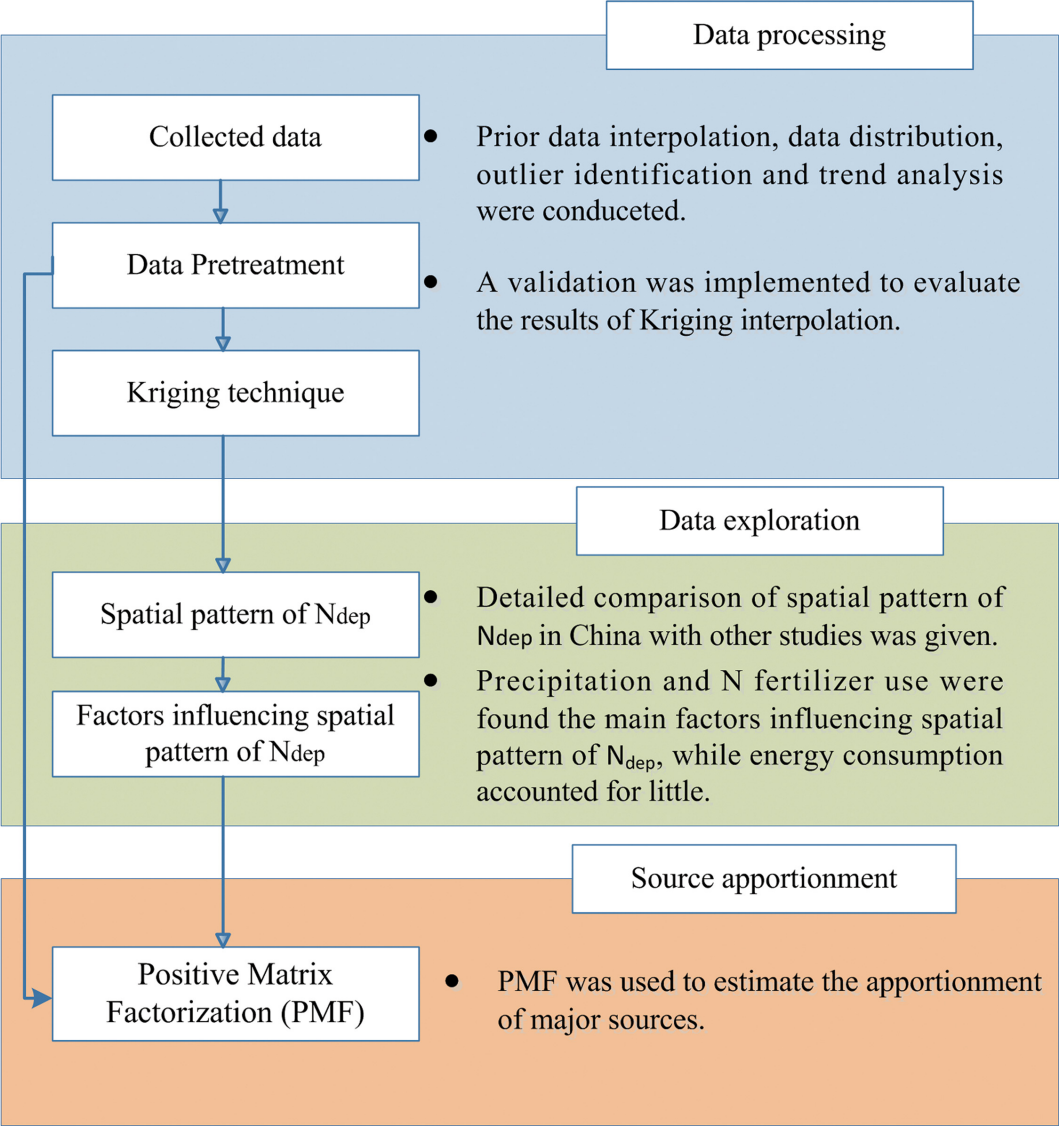
The flowchart of this study.

The flowchart of this study is shown in Fig 1. Firstly, the N fluxes from the published papers throughout China were obtained, and then the Kriging interpolation technique is applied to calculate N_dep_ on a national scale and compared the result with other N_dep_ maps in other studies. Then, the influence of P, F_N_ and E on the spatial pattern of N_dep_ is analyzed. Finally, potential sources of N_dep_ are evaluated.

### Data collection

**Fig 2:**
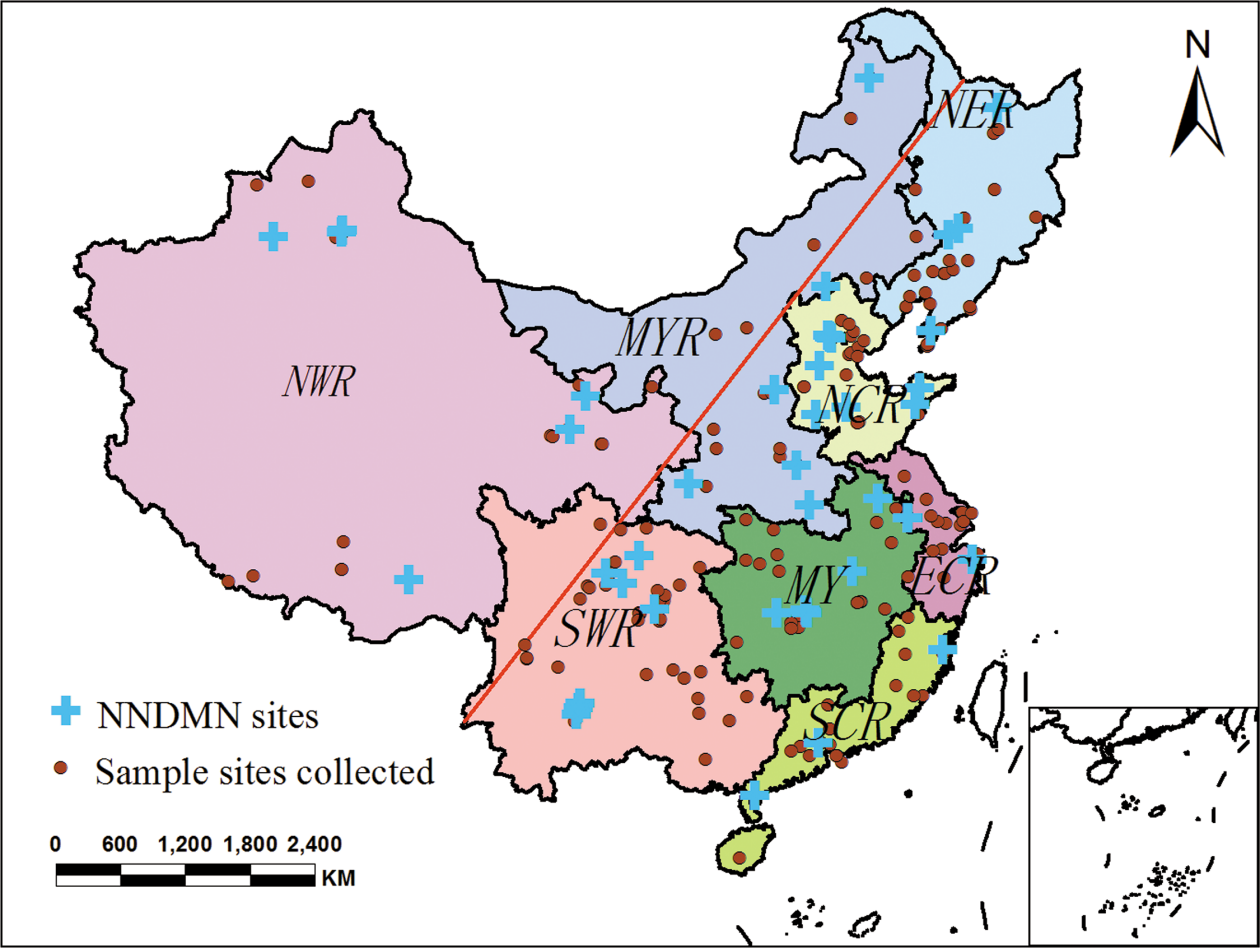
Spatial distribution of data points for N_dep_ in China (NER: Northeast region; NCR: North coastal region; ECR: East coastal region; SCR: South coastal region; MYR: Middle Yellow River; MY: Middle Yangtze; SWR: Southwest region; NWR: Northwest region). The red line divides China into a developed area (East) and an undeveloped area (West) in view of the levels of economic development, resource consumption, and population [5]. The NNDMN sites are from Nationwide Nitrogen Deposition Monitoring Network (NNDMN), organized by China Agricultural University [30].

To evaluate N_dep_ throughout China, it is critical to systematically collect the relevant published papers. In this study, the data pairs on precipitation sampling in China during 2003–2014 were collected. These studies were located by making a search through ISI Web of Knowledge using keywords “nitrogen deposition”, “chemical composition” or “precipitation” and “China”, and through CNKI website using the same Chinese keywords. Finally, 139 peer reviewed articles consisting 225 data records (Fig 2) on NH_4_^+^-N and NO_3_^-^-N in precipitation throughout China were collected (S1 Table). Basic information included the name of the monitoring sites, location, land use, rainfall, monitoring time span, annual precipitation, concentration and depositions of NH_4_^+^-N and NO_3_^-^-N and literature source from each study. To assure the monitoring quality of rainwater components, the studies based on the technical specifications required for acid deposition monitoring in China (State Environmental Protection Administration of China, 2004) were selected to establish datasets on N_dep_.

The data on the amount of F_N_ and E on provincial scales could be obtained from the China Statistical Yearbook from 2003 to 2014 (http://www.stats.gov.cn/tjsj/). Due to the lack of energy data in Tibet province, we assumed that the per capita energy consumption was similar between the Tibet and Xinjiang provinces, which are both located in western China, and deduced data on energy consumption in Tibet province from the Xinjiang province data.

The data on the annual precipitation were obtained from China Meteorological Administration. The mean annual precipitation in provinces was calculated based on the annual precipitation from 2003 to 2014, respectively, from the weather stations in each province.

### Calculation of wet N_dep_

Wet inorganic N deposition is calculated as the product of the precipitation amount and the concentration of N species in precipitation. The wet N deposition flux was kg N ha^-1^ and the unit of the precipitation is mm. The units of the concentration of N species in precipitation include mg N L^-1^ [30] and μeq L^-1^ [31]. Both of the two units are commonly used. Thus, when the unit of the concentration of N species is mg N L^-1^, the calculation formula of nitrogen deposition is:

$${\text{N}}_{\text{dep}}=\frac{C_{i}\times P_{i}}{100}$$ (1)

where N_dep_ is the N deposition flux per year (kg ha^-1^ a^-1^); *C*_*i*_ is the concentration of NH_4_^+^-N or NO_3_^-^-N (mg N L^-1^); *P*_*i*_ is the annual precipitation (mm); 100 is the conversion factor.

Otherwise, the formula is:

$${\text{N}}_{\text{dep}}=\frac{C_{i}\times P_{i}*14}{10^{5}}$$ (2)

where N_dep_ is the N deposition flux per year (kg ha^-1^ a^-1^); *C*_*i*_ is the concentration of NH_4_^+^-N or NO_3_^-^-N (μeq L^-1^); *P*_*i*_ is the annual precipitation (mm); 14 is the atomic weight of N and 10^5^ is the conversion factor.

### Geo-statistical method

A geostatistical method was used to produce spatially continuous estimates from discrete data points. National-scale N_dep_ maps were constructed using the Kriging interpolation technique. An unknown value associated with a point can be estimated by Kriging as follows:

$$Z\left(x_{0}\right)=\sum_{i=1}^{n}\;\lambda_{i}Z\left(x_{i}\right)$$ (3)

where *λ*_*i*_ is the Kriging weights computed from a normal system of equations using a semivariance function, derived by minimization of the error variance; the unknown value *Z*(*x*_*0*_) is interpreted as a random variable located in *x*_*0*_, as well as the values of neighbor samples *Z*(*x*_*i*_), *i* = 1, …, N.

Prior to Kriging interpolation, the Explore Data tool of ArcGIS 10.0 software is applied to conduct a data analysis, including data’s distributing, outlier identification, and trend analysis; the optimal variogram model and parameters are determined by GS plus.

### Source apportionment of ionic species

Positive matrix factorization (PMF) developed by the U.S. Environmental Protection Agency (EPA) is a multivariate factor analysis that utilizes error estimates and produces non-negative results [32]. PMF is used to factorize a given dataset into two matrices, the source profile (F) and source contribution (G), also called factors, which is expressed by the following formula:

$$x_{ij}=\sum_{k=1}^{p}g_{ik}f_{kj}+e_{ij}\;i=1,\ldots \;,m;j=1,\ldots \;,n;k=1,\ldots \;,p$$ (4)

where *x*_*ij*_ is are the elements of the input data matrix, *g*_*ik*_ and *f*_*kj*_ are the elements of the factor scores and factor loading matrices, respectively; *e*_*ij*_ is the residuals (i.e. the difference between input data and predicted values) and *p* is the number of factors resolved [33]. The resolving algorithm computes G and F elements that minimize the so-called object function Q.

$$\text{Q}=\sum_{i=1}^{m}\sum_{j=1}^{n}{\left[\frac{x_{ij}-\sum_{k=1}^{p}g_{ik}f_{kj}}{S_{ij}}\right]}^{2}$$ (5)

where *S*_*ij*_ represents the elements of uncertainty matrix, and each element is the uncertainty of *j*th species for sample *i*.

## Results and Discussions

### Accuracy assessment of the spatial N_dep_ distribution and comparisons with other studies

Although there were several studies on the estimation of wet N_dep_ on a national scale in China, most of them showed different spatial patterns. Which map of N_dep_ could reflect the real spatial distribution of N_dep_ in China is still a question.

**Fig 3:**
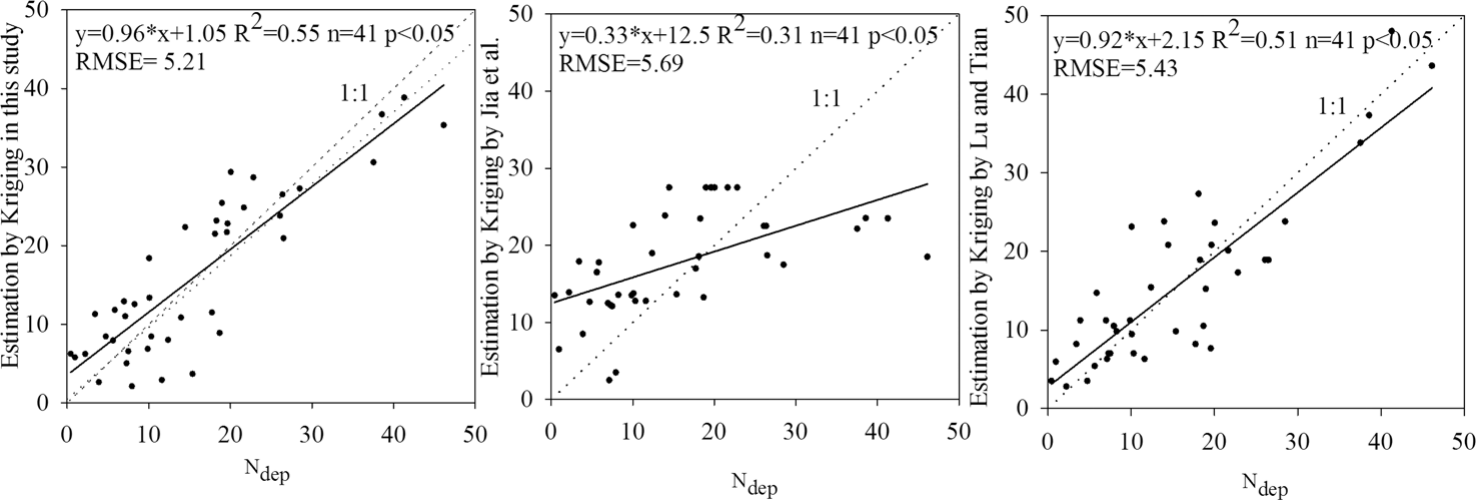
Comparison of N_dep_ (kg ha^-1^ a^-1^) monitored in 41 sites with the estimation results in this study, by Jia et al. [25] and Lu and Tian [14] (x-axis was the testing data in the work by Zhu et al. [4], y-axis was the results estimated in this study (a), by Jia et al. [25] (b), Lu and Tian [14] (c)). **Note**: a regression cofficient closer to 1.00, a higher R^2^ value indicate more reliable results of interpolation.

At a point scale, the 41 sites of N_dep_ in Zhu [4] were used to estimate the accuracy of the spatial distribution of N_dep_ by the method of Kriging. The Q-Q plot of the distribution of site-monitored N_dep_ versus that of the interpolated N_dep_ in this study is shown in Fig 3. The interpolated N_dep_ were distributed around the 1:1 line. The regression model between the original and interpolated N_dep_ had the regression coefficient (0.96) closer to 1 and a high R^2^ value. This indicated that there were close distributions between interpolated N_dep_ values and true N_dep_ values for the 41 testing data. The Q-Q plot of the N_dep_ from Zhu et al. [4] and Lu and Tian [14] versus the 41 testing data were also described in Fig 3. The N_dep_ by Lu and Tian [14] also obtained high accuracy, with low RMSE and high R^2^ values.

**Fig 4:**
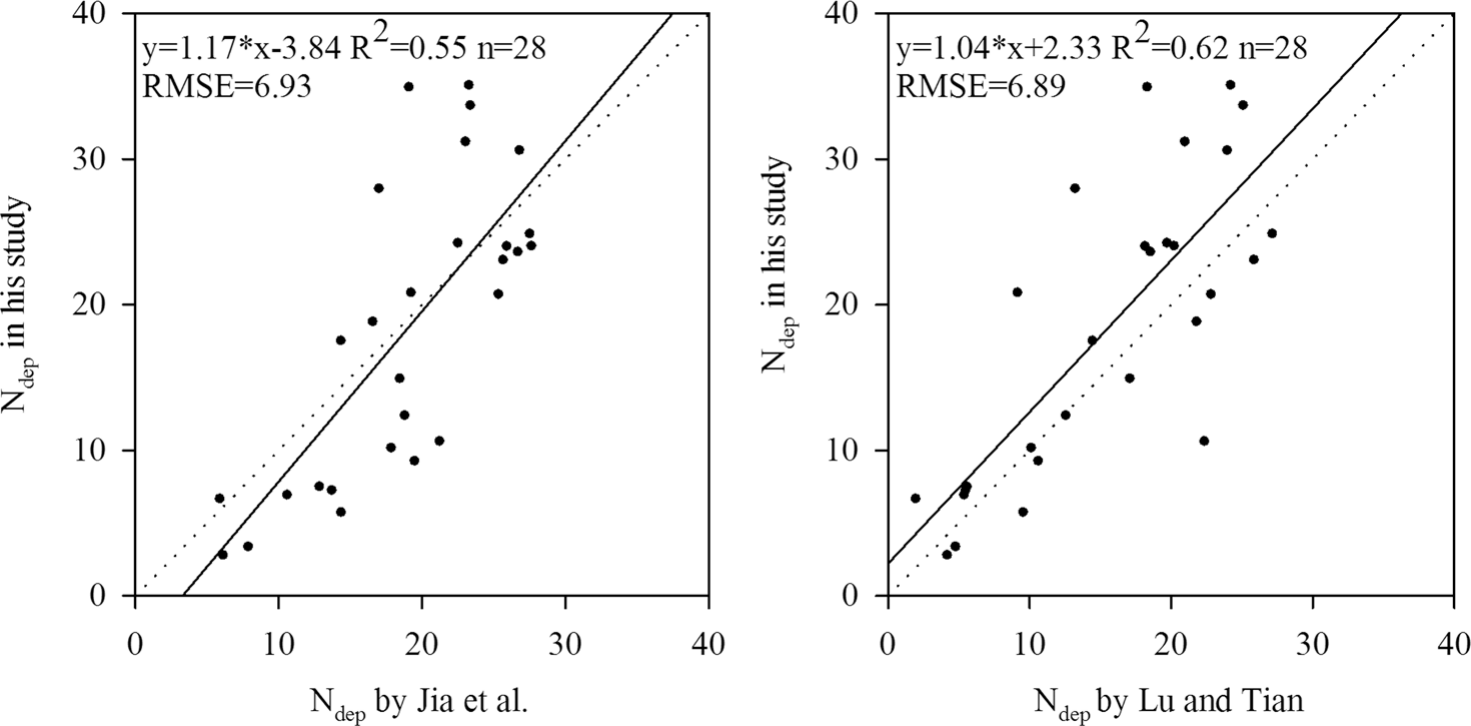
Comparison of N_dep_ (kg ha^-1^ a^-1^) with the results by Jia et al. [25] and Lu and Tian [14] at a provincial scale. **Note**: a regression cofficient closer to 1.00, higher R^2^ and small RMSE values indicate more reliable results of interpolation.

On a provincial scale, comparison of the results of N_dep_ (kg ha^-1^ a^-1^) in this study with those by Jia et al. [25] and Lu and Tian [14] is shown in Fig 4. Good agreements were also found for the comparison of N_dep_ with the results by Lu and Tian [14], giving confidence in the analysis of spatial pattern of N_dep_ in China. This also confirmed that our results were more consistent with that by Lu and Tian [14] than that by Jia et al. [25].

**Fig 5:**
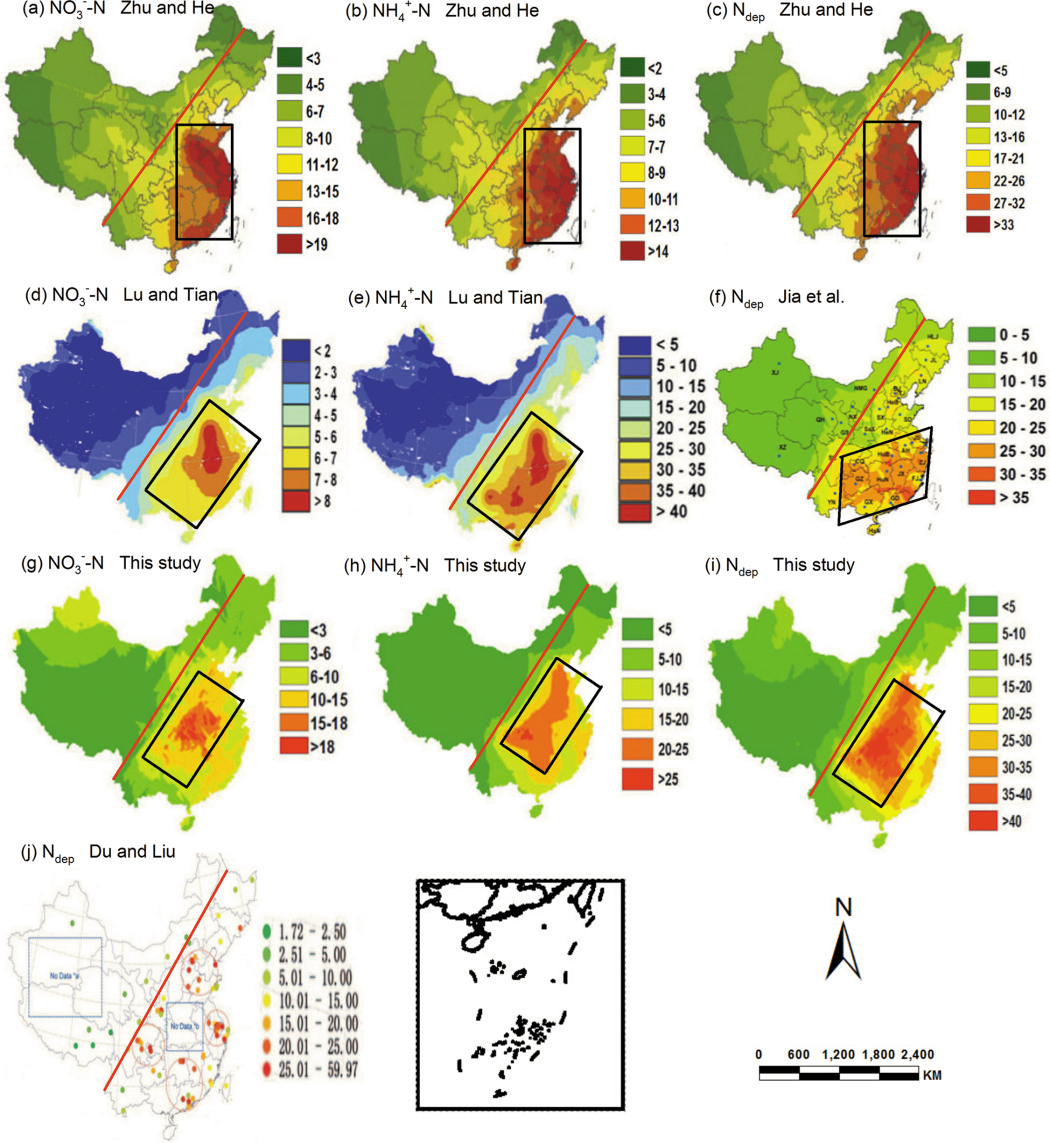
Spatial pattern of N_dep_ (kg ha^-1^ a^-1^) in China. Spatial distribution maps of N_dep_ between 2003 and 2014 were obtained from 182 monitoring sites by Kriging interpolation (g, NO_3_^-^-N; h, NH_4_^+^-N; i, total inorganic N) in this study, from 144 monitoring sites (f, total inorganic N) between 2000 to 2010 by Jia and Yu [25], from 41 sites (a, NO_3_^-^-N; b, NH_4_^+^-N; c, total inorganic N) in 2013 by Zhu and He [4], from 74 sites (j, total inorganic N) between 1995 and 2007 by Du and Liu [34], combining field measurements and monitoring estimating between 2000 to 2008 (d, NO_y_-N; e, NH_x_-N) by Lu and Tian [14]. The red line divides China into a developed area (East) and an undeveloped area (West) in view of the levels of economic development, resource consumption, and population [5].

On a national scale, to further explore the accuracy assessment of the spatial N_dep_ distribution, we compared our results with that by Lu and Tian [14] using the data of provided 74 monitored sites by Du and Liu [34] (Fig 5J). There were four hotspots on the N_dep_ map in this study, namely the North China Plain or Jing-jin-ji region, the Yangtze River Delta, Sichuan Basin and the Pearl River Delta. We suspected that Lu and Tian had underestimated slightly in Jing-jin-ji region, which should have the considerable magnitude of N_dep_ with three other hotspots (Fig 5J). However, Du and Liu [34] could not determine the magnitude of N_dep_ in Middle Yangtze region including Anhui province and in the south of Middle Yellow region including Henan province due to no data monitored. The work by Lu and Tian [14] reported this region also had high N_dep_ and we confirmed this hotspot in our study.

It should be noticed that this study might overestimate the N_dep_ on a national scale, since most of the monitoring sites used in these published papers in China were distributed in developed areas, which would overestimate N_dep_ on a national scale [5]. Also, there are some uncertainties in the estimation of N_dep_ in China, which resulted from different concepts, sampling procedures, analysis methods and scaling-up methods. The effects of scaling-up method on national scale results require further study and the observation network for N_dep_ needs to be strengthened to decrease the uncertainty.

### Spatial pattern of N_dep_ in China

The average of wet deposition flux of NH_4_^+^-N was 6.83 kg ha^-1^ a^-1^ with a standard deviation (STDEV) of 5.15, while the NO_3_^-^-N was 5.35 kg ha^-1^ a^-1^ with a STDEV of 5.71. The average of ratio of NH_4_^+^-N/NO_3_^-^-N was 1.28, which was slightly higher than the averaged ratio (1.22) in China, concluded by Zhu et al. [4]. The ratio of NH_4_^+^-N/NO_3_^-^-N was widely considered a proxy for the sources of atmospheric reactive N [4, 35, 36]. Agricultural activity is the main source of N_dep_ if the ratio is higher than 1, whereas, industrial activity is the main source if this ratio is lower than 1. The ratio of NH_4_^+^-N/NO_3_^-^-N in this study indicated both the agricultural and industrial activities collectively influence the deposition of atmosphere N.

The N_dep_ was 12.18 kg ha^-1^ a^-1^ and the total N deposition in China would be 20.30 kg ha^-1^ a^-1^ assuming that the contribution of dry deposition was about 40% in China [4, 37]. The magnitude and spatial pattern of N_dep_ differed significantly in different regions in China (Fig 5I). Both NH_4_^+^-N and NO_3_^-^-N peaked in central southern and southeastern China which are characterized by rapid industrial development and intensive N fertilizer applications [14]. N_dep_ exhibited a decreasing gradient from the southeast to the northwest of China. The red line (Fig 5) indicated the significant heterogeneity in the levels of economic development for different regions, which resulted in a matching spatial heterogeneity in N_dep_ across China. Similar results were also found in the study by Jia and Liu [3, 25]. The low N_dep_ were in areas including Qinghai-Tibet Plateau, Inner Mongolia and northwest China, where had not well developed industrial activities [5].

High N_dep_ occurred across the south of Middle Yellow region, the North Coastal region and the middle and lower reaches of Yangtze River Basin (Fig 5G, 5H and 5I), which was in good agreement with the results by Lu and Tian (Fig 5D and 5E), but much different with that by Jia et al. (Fig 5F) [14, 25]. Jia et al. [32] did not found the hotspots of N_dep_ in the south of Middle Yellow region including Henan and Shaanxi provinces and in the North Coastal region including Beijing, Tianjin, Hebei and Shandong provinces. Du and Liu [34] also concluded high N_dep_ in the North Coastal region including Beijing, Tianjin, Hebei and Shandong provinces (Fig 5J) [34] in good agreement with our findings. Jia et al. [25] maybe have underestimated N_dep_ in the North Coastal region due to the uncertainty resulting from the limited number of data and analysis method in this area. Liu et al. [26] believed that Zhu et al. [4] (Fig 5A, 5B and 5C) might underestimate the dissolved N deposition throughout China due to the uncertainty from limited number of samples (41 sites), and the storage in their studies [26]. This study also confirmed that Zhu et al. underestimated N_dep_ in the Southwest region including Chongqing and Guizhou provinces and the results by Du and Liu, Lu and Tian confirmed this suspect.

In summary, there were five hotspots of N_dep_ in China, including the North Coastal region, East Coastal region, Southwest region and South Coastal region, and Middle Yangtze. N_dep_ exhibited a decreasing gradient from southern to western and to northern China. N_dep_ was > 35 kg ha^-1^ a^-1^ in some provinces of southern China, such as Chongqing, Hunan, Hubei and Henan, whereas N_dep_ in other provinces of southern China was about 20–35 kg ha^-1^ a^-1^. N_dep_ over northern, northeastern and northwestern China was about 10–20, 5–15, 0–10 kg ha^-1^ a^-1^.

The N_dep_ on a national scale ranged from 9.88 to 21.1 kg ha^-1^ a^-1^ (Table 1), showing strong spatial variations. The wet deposition flux of N_dep_ (12.18 kg ha^-1^ a^-1^) in this study was much lower than that (21.07 kg ha^-1^ a^-1^) based on the average of those data points to represent N_dep_ status across the whole China [3]. It was a bit higher than that (9.88 kg ha^-1^ a^-1^) by Lu and Tian (2007) calculated from at 253 sites from 1990 to 2003, and it was close to the results by Jia et al. (13.87 kg ha^-1^ a^-1^), Lu and Tian (14.05 kg ha^-1^ a^-1^) and Zhu (13.18 kg ha^-1^ a^-1^). These similar studies all considered spatial variability and area-weighted contribution from high- and low-N deposited regions, which was critically important to generate estimation of N_dep_ on a national scale [6, 14].

**Table 1:** Atmospheric N deposition (kg ha^-1^ a^-1^) on the bias of different methods and temporal scales.

Estimation technique	Year	NH_4_^+^-N	NO_3_^-^-N	N_dep_	Reference
Summarized previous results	2000–2010	-	-	21.1^b^	[3]
Data collection	2000–2010	-	-	13.87	[25]
Combining measurements and estimating	2000–2008	-	-	14.05^a^	[14]
Measurements	2013	7.25	5.93	13.18	[4]
Measurements	1995–2007	10.66 ^b^	6.57 ^b^	17.36 ^b^	[34]
Measurements	1990–2003	-	-	9.88	[1]
Summarized previous results	2003–2014	6.83	5.35	12.18	This study

^a^Given the ratio of wet to bulk N deposition (20.07) as 0.7, the wet N deposition was 14.05.

^b^The averaged value ignoring difference between regions.

### Influencing factors of Precipitation (P), N fertilizer use (F_N_) and energy consumption (E) on the spatial patterns of N_dep_

The process of N_dep_ is relatively clear in theory and has been applied in models, however, no agreement was reached upon how P, F_N_ and E inflenced N_dep_. It is critical to understand the realationship between N_dep_ and P, F_N_ and E, to simulate and predict future trends in N_dep_ assuming that the existing emission factors for F_N_ and E don't change much.

Several models have been developed to simulate the correlation of N_dep_ and P, F_N_, E (Table 2). Jia et al. found that N_dep_ was linearly related to P and logarithmically to F_N_ and E [25]. They believed that E, F_N_ and P should be considered together when studying the factors that control the spatial pattern of N_dep_ on the regional scale. N_dep_ was calculated using equation N_dep_ = a*ln((F_N_*18.5%+E*0.24%)*P)+b. However, Zhu and He reported N_dep_ was exponentially related to P and E and linearly related to F_N_ [4]. They thought that P and F_N_ explain 80%-91% of the spatial variation of N_dep_, whereas E did not significantly explain the variability. A multiple linear regression model (N_dep_ = a+b*F_N_+c*P) was applied without E by Zhu and He.

**Table 2:** Comparison of different models used to simulate P, F_N_ and E influencing spatial patterns of N_dep_

N_dep_ and P, F_N_ and E	N_dep_ and P	N_dep_ and F_N_	N_dep_ and E	Reference
N_dep_ = a*ln((F_N_*18.5%+E*0.24%)*P)+b	y = a*P+b	y = a+b*ln(F_N_)	y = a+b*ln(E)	[25]
N_dep_ = a+b*F_N_+c*P	y = a*P^b^	y = a* F_N_+b	y = a*E^b^	[4]
-	y = a*P+b	y = a+b*ln(F_N_)	y = a+b*ln(E)	[38]
N_dep_ = a+b*F_N_^c^+d*P^e^	y = a*P^b^	y = a*F_N_^b^	y = a*E^b^	This study

a, b, c, d, e are regression coefficients; N_dep_ represents N deposition; P represents precipitation (mm); F_N_ represents fertilizer N use (t km^-2^ a^-1^); E represents energy consumption (t km^-2^ a^-1^).

**Fig 6:**
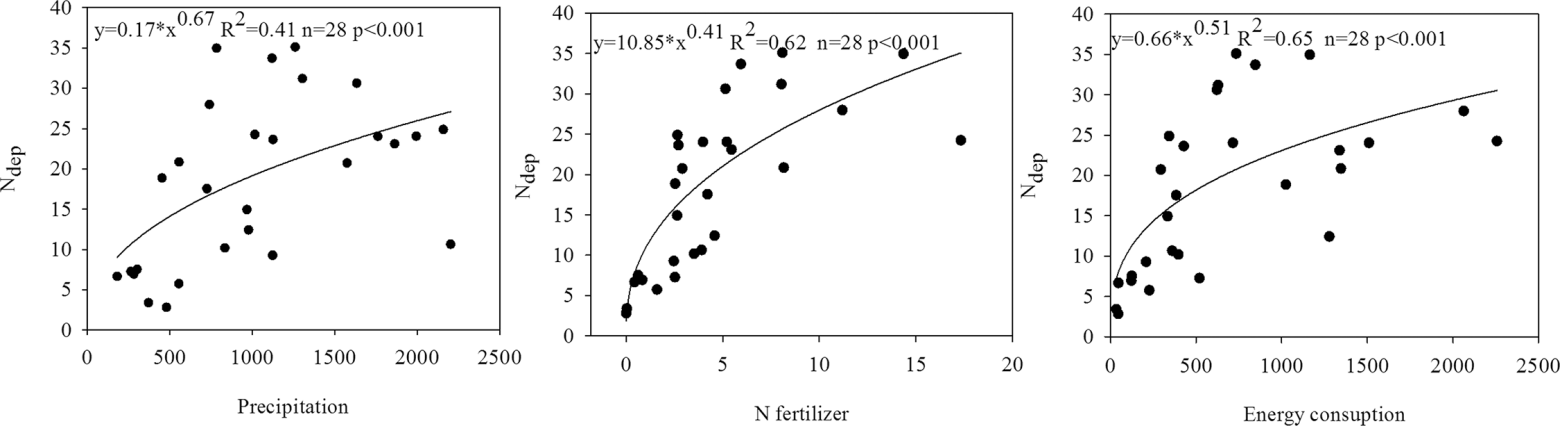
The effects of precipitation (mm), N fertilizer (t km^-2^ a^-1^) and energy consumption (t km^-2^ a^-1^) on the spatial pattern of N_dep_ (kg ha^-1^ a^-1^). The mean N_dep_ (kg ha^-1^ a^-1^) in provinces were obtained from spatial maps of N_dep_ (kg ha^-1^ a^-1^) in China using Kriging.

**Fig 7:**
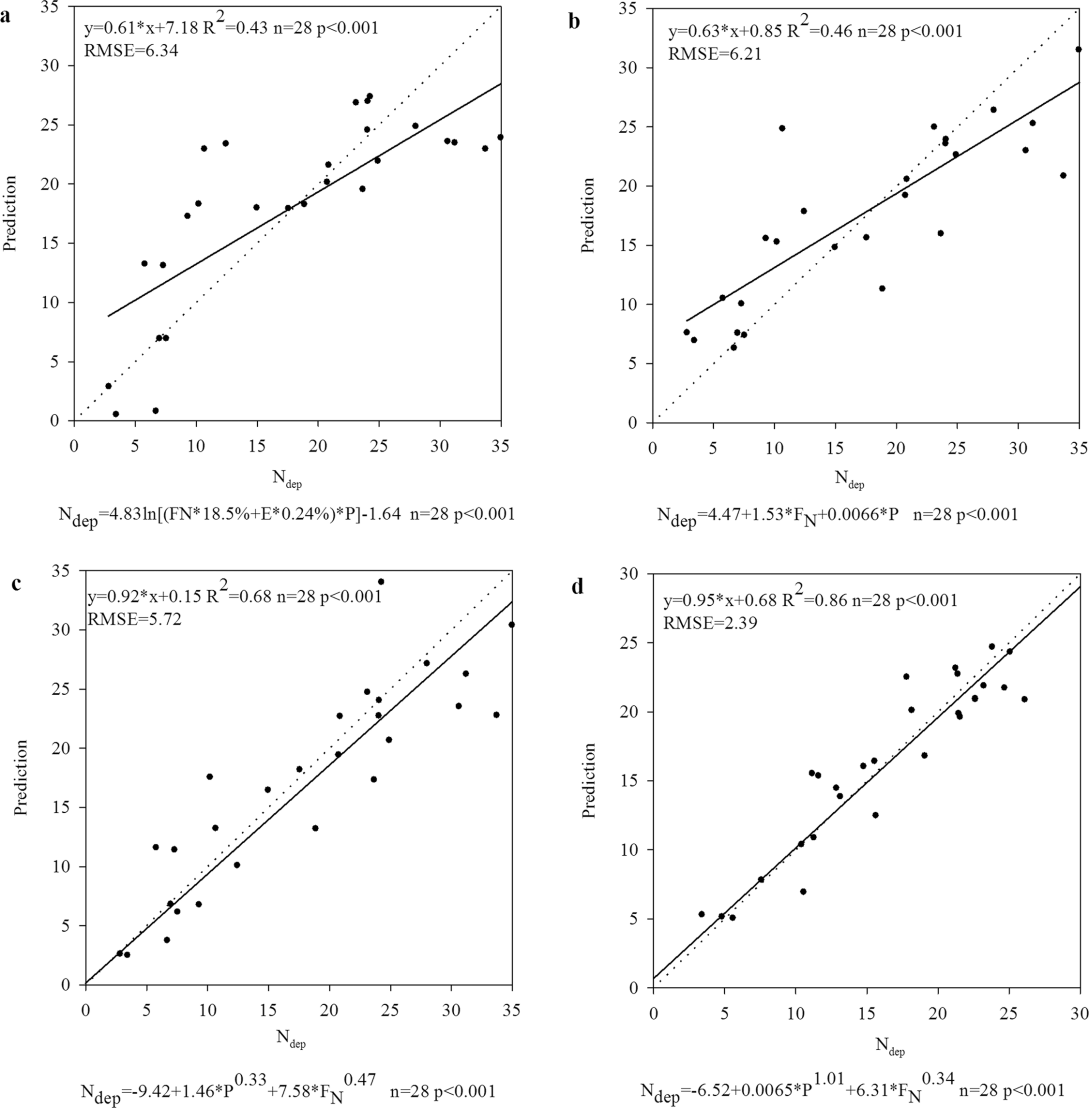
Test of equations using data from 2003 to 2014. The x-axis variable (this study: a, b, c; Jia et al. [25]: d) was the modeled results of N_dep_ (kg ha^-1^ a^-1^) in provinces as obtained by Kriging method and data on precipitation (mm), N fertilizer (t km^-2^ a^-1^) and energy consumption (t km^-2^ a^-1^) in provinces excluding Beijing, Shanghai and Tianjin. The y-axis variable was calculated by different prediction model equations (Jia et al. [25] (a): N_dep_ = a*ln((F_N_*18.5%+E*0.24%)*P)+b; Zhu and He [4] (b): N_dep_ = a+b*F_N_+c*P; this study (c, d): N_dep_ = a+b*F_N_^c^+d*P^e^). **Note**: a regression cofficient closer to 1.00 and higher R^2^ and small RMSE values indicate more reliable results. The regression cofficient reached approximately 0.92 and R^2^ were about 0.58 in this study.

In this study, a strong exponential correlation was found between P and N_dep_, F_N_ and N_dep_, E and N_dep_ (Fig 6), which was in good agreement with that conducted by Zhu and He [4]. The models by Jia et al. (Fig 7A) and Zhu and He (Fig 7B) were applied to predict N_dep_ in China in this study. To improve this estimation of N_dep_, we established a new model to simulate this correlation based on a strong exponential correlation found (Fig 6). We agreed that E had little effect on the spatial pattern of N_dep_ proposed by Zhu and He [4] through our practice in this study. Thus, we adopted an equation (N_dep_ = a+b*F_N_^c^+d*P^e^) to predict N_dep_ and found a higher R^2^ (Fig 7C) compared with the results by Jia et al. (Fig 7A) and Zhu and He (Fig 7B). To confirm the effective of this new model, we used the data published by Jia et al. [25] to test whether this equation can reflect the spatial variation of N_dep_ in China in 2000s and good agreement was found for the comparison of N_dep_ with prediction (Fig 7D).

It should be noted that we agree with E contributing much to the magnitude of decadal N_dep_ in China [25], but had little effect on the spatial variation of N_dep_ [4]. In summary, P, F_N_ and E were all significantly correlated with the magnitude of N_dep_, P and F_N_ contributed more than E to the spatial variation of N_dep_. It was critically essential to reduce E and F_N_ to control reactive N emissions from fossil fuel combustion using maximum fessible reduction [4, 22].

Ceratianly, we had to admit that there were some uncertainties in the analysis of how P, F_N_ and E influencing the spatial patterns of N_dep_, which resulted from the limited statistical data obtained. The constructed analytical relationship was based on a provincial statistical data, and we believe that more data, such as municipal or county-level data, will obtain more reliable statistical models. However, it was too difficult to obtain such municipal or county-level data on both F_N_ and E from the statistical yearbooks in China. The data on energy consumption (expressed as standard coal) on a municipal or county-level scale were not included in municipal or county-level statistical yearbook and only the total energy consumption on a provincial scale could be obtained. Thus, we have to use the provincial statistical data to explore the correlation.

### Anthropogenic sources of N_dep_ in China

**Fig 8:**
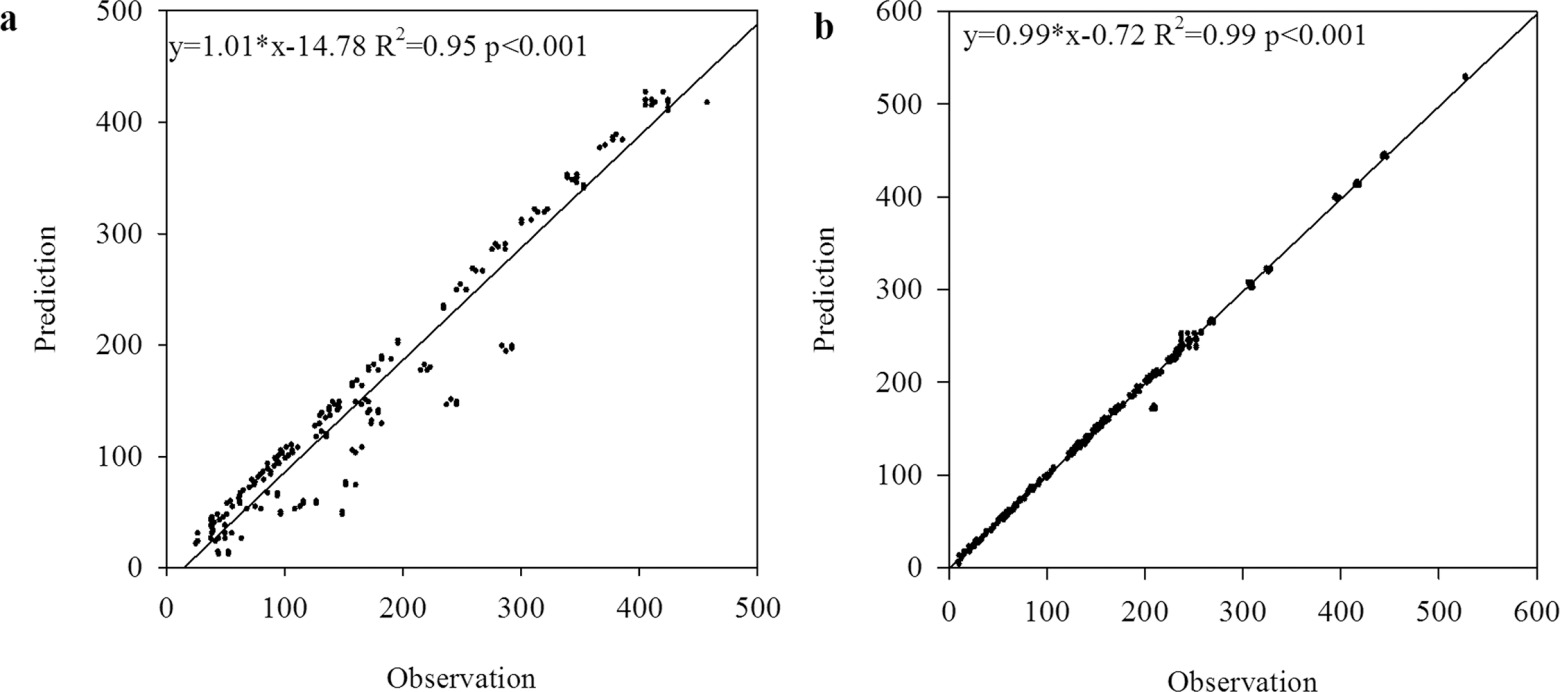
Comparison of PMF predictions with observations for NO3- (a) and NH4+ (b) concentrations (μeqL^− 1^) in the wet deposition samples from 2003 to 2014 in China.

Detailed source contributions data are critical for policy makers to develop effective policies to protect Chinese terrestrial ecosystems [3]. Fossil fuel combustion and agricultural activities were likely the main anthropogenic sources for NH_4_^+^-N and NO_3_^-^-N depositions, but their relative contributions in China cannot be determined in previous studies. In this study, a PMF source apportionment analysis was used to further explore the main source of N_dep_. Fig 8 shows a comparison of the observed and PMF predicted concentration of NO_3_^-^ and NH_4_^+^ for each sample. Excellent agreement was found, giving confidence that the PMF model captured the major sources and correctly quantified their contributions.

**Fig 9:**
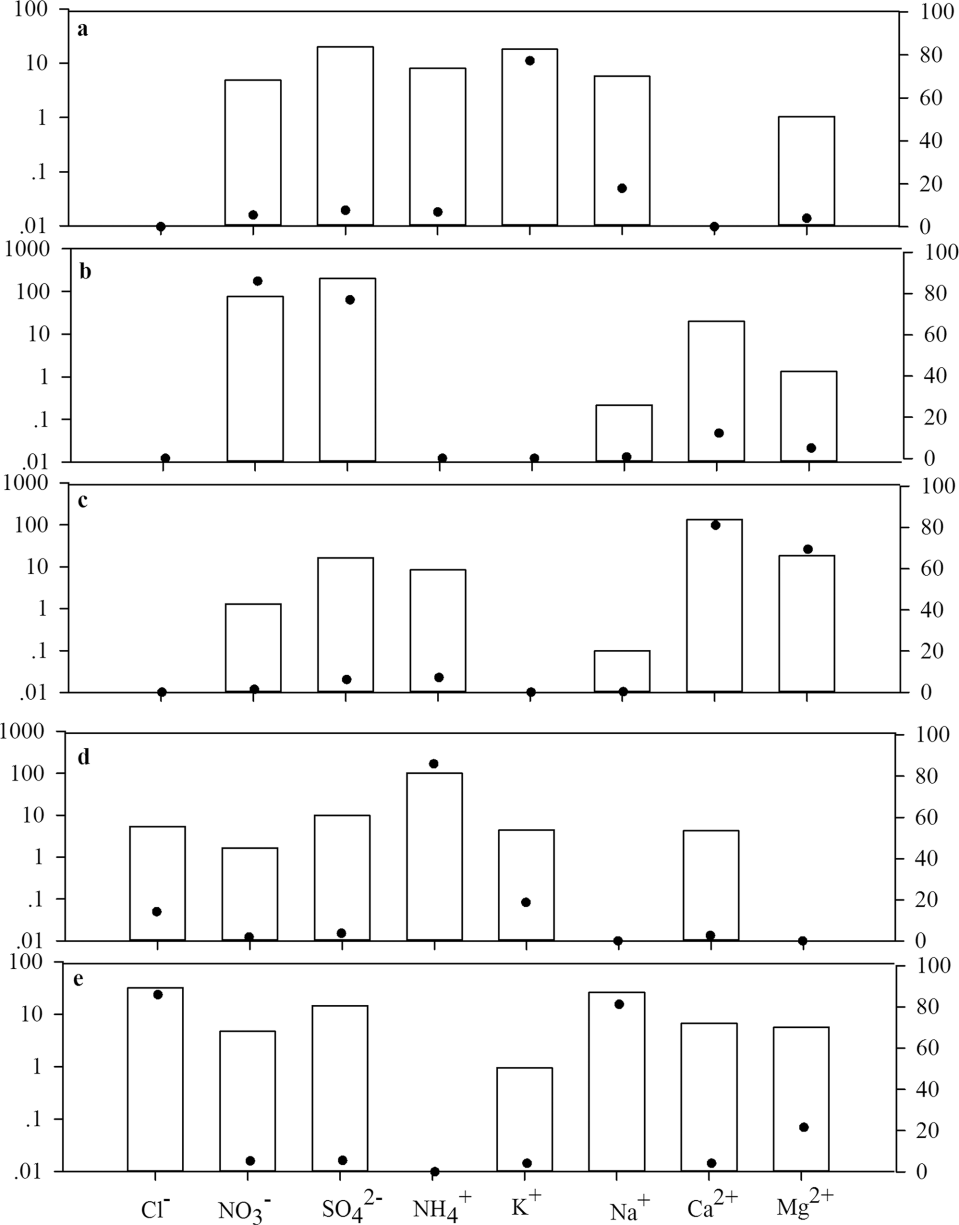
Predicted source profiles of PMF for wet deposition data collected in China. The bars indicate source profiles (left y-axis), and the filled dots indicate percentage of species (right y-axis) attributed to that source. (Biomass burning (a), Fossil fuel combustion (b), Crust (c), Agriculture (d), Aged sea salt (e).).

The PMF model resolved five distinct sources (Fig 9). The first source had high K^+^, indicating a biomass burning (Fig 9A). The second source was enriched with SO_4_^2-^ and NO_3_^-^ (Fig 9B), indicating a fossil fuel combustion source. The two icons were associated with NO_x_ emitted from coal-fired power plants, residential heating and cooking, and motor vehicles [39]. The third source had a high loading of Ca^2+^ and Mg^2+^, representing a crustal or windblown dust source (Fig 9C). The profile also contained a significant SO_4_^2-^ indicating a great effect of neutralizing the acid [39]. The fourth source was dominated by NH_4_^+^ suggesting an agricultural source (Fig 9D). The fifth source had high loading of Na^+^ and Cl^-^, a clear signal of sea salt impact (Fig 9E). However, the profile also contained a significant SO_4_^2-^, a typical characteristic of aged sea salt.

**Fig 10:**
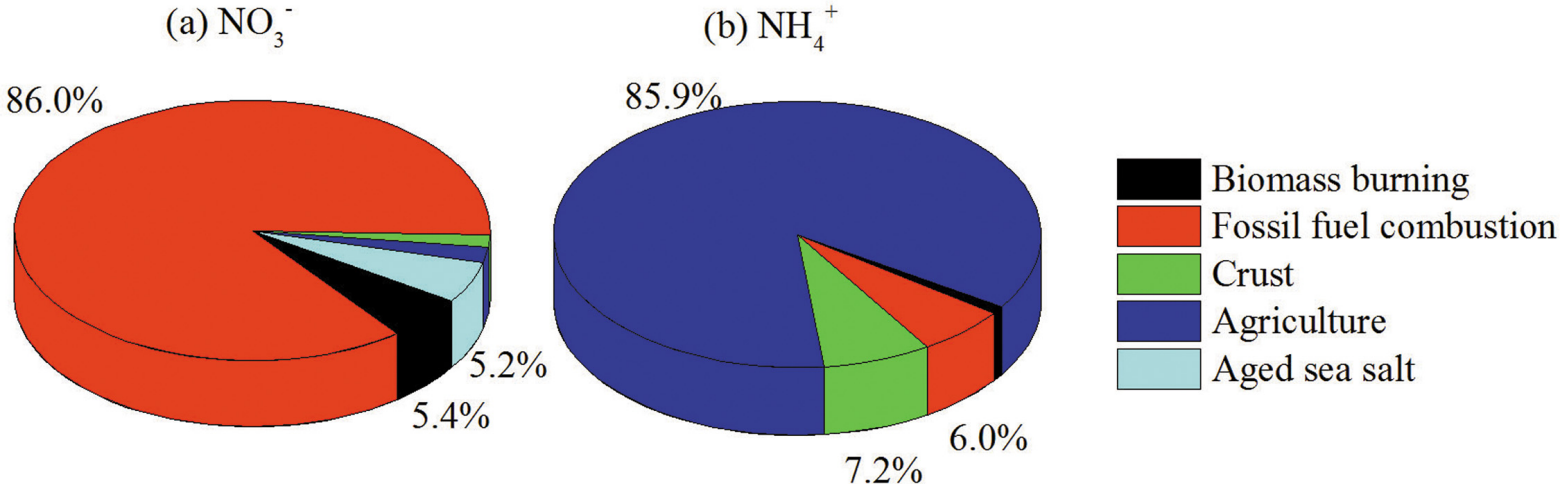
Percentage contributions of aged sea salt, crust, agriculture, fossil fuel combustion, and biomass burning to annual wet deposition flux of NH_4_^+^-N, NO_3_^-^-N in China between 2003 and 2014.

The percentage contributions of each source to NH_4_^+^-N and NO_3_^-^-N are shown in Fig 10. Fossil fuel combustion was the main contributor to NO_3_^-^-N (86.0%). Biomass burning also contributed to 5.4% on the deposition of NO_3_^-^-N. NH_4_^+^-N was mainly from agricultural activities (85.9%), fossil fuel combustion (6.0%) and Crust (7.2%). Overall, N_dep_ in China may be considerably affected by the high emissions of NO_x_ and NH_3_ from fossil fuel combustion and agricultural activities and relevant studies will be presented in future papers.

## Conclusion

The N_dep_ throughout China was obtained by a method of Kriging, based on the N fluxes from the published papers from 2003 to 2014. The N_dep_ map in our study showed close spatial pattern with that by Lu and Tian (2014). There were five hotspots of N_dep_ across the North Coastal region, East Coastal region, Southwest region and South Coastal region, and Middle Yangtze, and exhibited a decreasing gradient from southeast to northwest of China. The wet deposition flux of NH_4_^+^-N, NO_3_^-^-N and total N_dep_ was 6.83, 5.35 and 12.18 kg ha^-1^ a^-1^, respectively. A strong exponential correlation was found between P and N_dep_, F_N_ and N_dep_ and E and N_dep_, P and F_N_ (80–91%) contributed more than E to the spatial variation of N_dep_. Fossil fuel combustion was the main contributor to NO_3_^-^-N (86.0%) and biomass burning also contributed to 5.4% on the deposition of NO_3_^-^-N. NH_4_^+^-N was mainly from agriculture (85.9%), fossil fuel combustion (6.0%). Our findings confirmed that the anthropogenic activities were the main reason for N_dep_ increase and provided a scientific background for studies on ecological effects of N_dep_ in China.

## Supporting Information

S1 PRISMA Checklist The PRISMA 2009 Checklist (DOC)

S1 Table The information of the collected data records in this study. (XLSX)
